# Methods for evaluating gene expression from Affymetrix microarray datasets

**DOI:** 10.1186/1471-2105-9-284

**Published:** 2008-06-17

**Authors:** Ning Jiang, Lindsey J Leach, Xiaohua Hu, Elena Potokina, Tianye Jia, Arnis Druka, Robbie Waugh, Michael J Kearsey, Zewei W Luo

**Affiliations:** 1 School of Biosciences, The University of Birmingham, Edgbaston Birmingham B15 2TT, England, UK; 2 Scottish Crop Research Institute, Invergowrie, Dundee DD2 5DA, Scotland, UK; 3 Institute of Biostatistics, Fudan University, Shanghai 200433, PR China

## Abstract

**Background:**

Affymetrix high density oligonucleotide expression arrays are widely used across all fields of biological research for measuring genome-wide gene expression. An important step in processing oligonucleotide microarray data is to produce a single value for the gene expression level of an RNA transcript using one of a growing number of statistical methods. The challenge for the researcher is to decide on the most appropriate method to use to address a specific biological question with a given dataset. Although several research efforts have focused on assessing performance of a few methods in evaluating gene expression from RNA hybridization experiments with different datasets, the relative merits of the methods currently available in the literature for evaluating genome-wide gene expression from Affymetrix microarray data collected from real biological experiments remain actively debated.

**Results:**

The present study reports a comprehensive survey of the performance of all seven commonly used methods in evaluating genome-wide gene expression from a well-designed experiment using Affymetrix microarrays. The experiment profiled eight genetically divergent barley cultivars each with three biological replicates. The dataset so obtained confers a balanced and idealized structure for the present analysis. The methods were evaluated on their sensitivity for detecting differentially expressed genes, reproducibility of expression values across replicates, and consistency in calling differentially expressed genes. The number of genes detected as differentially expressed among methods differed by a factor of two or more at a given false discovery rate (FDR) level. Moreover, we propose the use of genes containing single feature polymorphisms (SFPs) as an empirical test for comparison among methods for the ability to detect true differential gene expression on the basis that SFPs largely correspond to *cis*-acting expression regulators. The PDNN method demonstrated superiority over all other methods in every comparison, whilst the default Affymetrix MAS5.0 method was clearly inferior.

**Conclusion:**

A comprehensive assessment of seven commonly used data extraction methods based on an extensive barley Affymetrix gene expression dataset has shown that the PDNN method has superior performance for the detection of differentially expressed genes.

## Background

Affymetrix GeneChip microarrays are the most popular high density oligonucleotide gene expression arrays and have become an invaluable tool in genomics studies worldwide. Each gene on an Affymetrix microarray GeneChip is typically represented by a probe set consisting of 11 different pairs of 25-bp oligos covering features of the transcribed region of that gene. Each pair consists of a perfect match (PM) and a mismatch (MM) oligonucleotide. The PM probe exactly matches the sequence of a particular standard genotype, often one parent of a cross, while the MM differs in a single substitution in the central, 13^th ^base. The MM probe is designed to distinguish noise caused by non-specific hybridization from the specific hybridization signal.

Affymetrix microarrays inevitably introduce many sources of variation [1]. Normalization procedures are essential to "correct" for systematic sources of variation of non-biological origin. Affymetrix microarray data are normalized in three steps: background correction, to adjust for hybridization effects unrelated to the interaction between probes and target DNA; normalization, to remove systematic errors and biases thereby allowing data to be compared from one array to another; summarization, combining the multiple probe intensities from a probe set to yield a single value for each gene that best represents the expression level of the RNA transcript. Numerous data extraction methods have been proposed in the literature to perform these crucial steps in processing Affymetrix oligonucleotide microarray data.

The first data extraction method provided as the Affymetrix default was the Average Difference (AD), a linear scale measure that relied upon the difference measure PM-MM to correct for non-specific binding. This measurement was superseded by the current standard MAS5.0 [2], which uses the more appropriate log scale and a robust Tukey Biweight averaging method. It was shown subsequently that one third of probe pairs consistently yield negative signals, showing that use of MM probes for detection of non-specific binding is unreliable [3,4]. In this respect, Irizarry et al. [5] developed the robust multi-array average (RMA) method based solely on PM values. Li and Wong [6] developed a statistical model for probe level data and their model based expression index (MBEI) has been developed into dChip, one of the most popular software approaches used today. Physical energy-based models have also been developed as an attempt to model the formation of DNA-RNA duplexes on oligonucleotide microarrays [7], most notably the positional dependent nearest neighbour (PDNN) model of Zhang et al. [8]. Following this idea, Wu et al. [9] developed the GCRMA method that attempts to combine the strengths of stochastic model based algorithms such as RMA with physical modelling of sequence information. The number of methods available continues to grow, yet there is no consensus as to which is the most appropriate and reliable method for a given application.

Calibration datasets derived from mixture experiments [10], spike-in studies and dilution series [3,5,11-14] have been an invaluable resource to develop and assess data extraction methodology. The advantage of these benchmarking datasets is that the expected outcome of expression analysis is known in advance and so alternative expression measures can be compared in terms of the expected features. This property has been exploited to develop a graphical tool for the evaluation and comparison of expression measures aimed at helping researchers to decipher the multitude of methods available [12,14].

Studies utilizing benchmark datasets have typically observed a large effect of the normalization method on the outcome of the expression analyses [15-17]. However, the performance of 'spike-in' experiments can be affected by sources of systematic variation and it is not clear how this might affect evaluation of different data extraction methods [15]. One alternative strategy involved assessing the gene expression between males and females at Y-chromosome linked genes as a true biological internal control [18]. In this study, the performance of the method was measured by recording how many differentially expressed Y-chromosome linked genes were detected between male and female samples. However, the general applicability of this kind of test is limited.

More recently, Harr and Schlotterer [15] introduced an alternative strategy to evaluate normalization methods by exploiting the existence of bacterial operons in which genes are expected to have highly correlated expression levels. This strategy effectively avoided the systematic biases inherent in the spike-in approach. However, the assumption that expression of operon member genes should be correlated can be violated, for example by internal promoters and/or overlapping regulatory elements [19]. It is increasingly evident that performance analyses using calibration datasets are not necessarily consistent with data from realistic biological studies [16,20], suggesting the need to consider real biological studies in an attempt to evaluate the relative merits of Affymetrix data extraction methods.

In this article we present a comparison of the influence of seven commonly used data extraction methods on the detection of differentially expressed genes using a genome-wide gene expression dataset from eight genetically divergent barley lines. The major challenge arising from the use of this dataset is that one has no *a priori *knowledge of which genes are differentially expressed. To address this challenge we used a novel strategy based on genes in which we detected single feature polymorphisms (SFPs). SFPs are genetic polymorphisms in observed expression within one particular feature (oligonucleotide probe) of a probe set (11 PM and MM probes) on the array [21]. Using two barley 'Genetical Genomics' datasets we have previously shown that SFPs mainly represent expression differences that are the result of polymorphism in *cis*-acting regulators [22]. On this basis we propose that differential expression detected in SFP-containing genes is more likely to reflect true differential expression and so we use this as a criterion to assess the efficacy of the seven methods referred to above in the detection of differential gene expression.

## Results

The present study implements seven methods commonly used in the literature to calculate expression indices from Affymetrix microarray gene expression data, which was collected from a well-designed genome-wide microarray hybridization experiment with eight genetically divergent barley cultivars. These methods are summarized in Table 1 and include Average Difference (AD), MAS5.0, MBEI (PM only), MBEI (PM-MM), RMA, PDNN and GCRMA. We explore various statistical properties of the methods in modelling and analyzing the microarray dataset. The findings are compared with those based on an independent dataset of Affymetrix genome-wide gene expression profiled on two divergent yeast strains.

**Table 1:** Statistical analyses involved in the seven different methods for calculating gene expression.

Methods	Background Correction	Normalization	Core Statistical Analysis	References
AD	None	Invariant Set	Average difference	Affymetrix [2]
MAS5.0	Spatial effect and MM subtracted	Constant	Robust average (Tukey bi-weight)	Affymetrix [2]
MBEI (PM only)	None	Invariant Set	Multiplicative model	Li and Wong [5]
MBEI (PM-MM)	MM intensities are subtracted	Invariant Set	Multiplicative model	Li and Wong [5]
RMA	Global correction	Quantile	Robust linear model (median polish)	Irizarry et al. [4]
PDNN	Model is fitted accounting for background and specific signal	Quantile	Specific and non-specific binding effects are estimated using free energy model	Zhang et al. [7]
GCRMA	Based on probe sequence	Quantile	Robust linear model (median polish)	Wu et al. [8]

### Consistency of gene expression indices calculated from different methods

To explore the consistency of the 22,840 barley gene expression indices estimated from the seven different methods, we calculated Pearson's Product Moment Correlation coefficients in the expression estimates and the correlation analyses are summarized in Table 2. The corresponding results based on the yeast dataset are summarized in Table 4 [see Additional file 1]. The upper triangle in Table 2 contains the means and standard deviations of 24 correlation coefficients, *r*_*ijk *_(*k *= 1, 2,..., 24). *r*_*ijk *_represents the correlation coefficient between 22,840 corresponding pairs of gene expression indices calculated by methods *i *and *j *from the *k*^th ^microarray sample. The lower triangle shows the overall correlation coefficients between all pairs of 22,840 gene expression indices calculated from methods *i *and *j *across all 24 samples (cultivars *m *= 1,...,8 × replicates *n *= 1,...,3). It is clear that the seven methods may be separated into two groups (AD, MAS5.0 and MBEI in one group and RMA, GCRMA and PDNN in the other) according to the correlation coefficients. The coefficient of correlation is greater than 90% within each of the groups but becomes less than 80% between the two groups. The same pattern of correlation in gene expression estimate between these seven methods was also recovered in the analysis of gene expression profiles on two yeast strains. Notably, all of the methods in the first group were based on use of both PM and MM values (with the exception of MBEI PM), while the methods in the second group were based on PM value only. However, the average correlation coefficient between MBEI PM and MBEI PM-MM was as high as 0.988, therefore the division of the seven methods into two groups was unlikely to be caused by using either the PM-MM model or the PM only model.

**Table 2:** Pearson's Product Moment Correlation Coefficients among barley gene expression indices calculated from seven different methods.

Method	AD	MAS5.0	MBEI^1^	MBEI^2^	RMA	GCRMA	PDNN
AD	**0.991 ± 0.005**	0.975 ± 0.004	0.988 ± 0.001	0.985 ± 0.001	0.647 ± 0.007	0.791 ± 0.008	0.615 ± 0.007
MAS5.0	0.973	**0.984 ± 0.009**	0.961 ± 0.005	0.965 ± 0.003	0.619 ± 0.026	0.748 ± 0.024	0.583 ± 0.026
MBEI^1^	0.987	0.958	**0.990 ± 0.005**	0.988 ± 0.001	0.664 ± 0.011	0.797 ± 0.009	0.629 ± 0.008
MBEI^2^	0.985	0.963	0.988	**0.990 ± 0.005**	0.643 ± 0.006	0.774 ± 0.006	0.605 ± 0.006
RMA	0.647	0.616	0.662	0.643	**0.993 ± 0.003**	0.914 ± 0.002	0.939 ± 0.008
GCRMA	0.791	0.744	0.797	0.774	0.914	**0.992 ± 0.005**	0.923 ± 0.004
PDNN	0.614	0.581	0.628	0.604	0.940	0.923	**0.992 ± 0.005**

The upper triangle shows the mean and corresponding standard deviation of 24 correlation coefficients, *r*_*ijk*_, (*k *= 1, 2,..., 24). *r*_*ijk *_represents the correlation coefficient between 22,840 corresponding pairs of gene expression indices calculated by methods *i *and *j *from the *k*^th ^microarray sample. The diagonal cells show means and standard deviations of 24 correlation coefficients, *r*_*imn *_(*n *= 1, 2, 3 and *m *= 1, 2,..., 8). For *n *= 1, 2, 3, *r*_*imn *_corresponds to three correlation coefficients calculated from three possible pairs of replicates for the *m*^th ^cultivar (*m *= 1, 2,..., 8) using method *i*. The lower triangle shows the correlation coefficients between all pairs of 22,840 gene expression indices calculated from methods *i *and *j *across all *k *= 24 samples.

^1 ^MBEI PM only model

^2 ^MBEI PM-MM model

The diagonal elements in Table 2 represent means and standard deviations of correlation coefficients in gene expression indices between biological replicates. They show that MAS5.0 confers significantly lower correlations between replicates than the other methods (*p*-value < 10^-5^, Mann-Whitney U-test), suggesting that the different methods have a profound effect that goes beyond the variance observed across the biological replicates, in support of previous findings [15,17].

**Figure 1:**
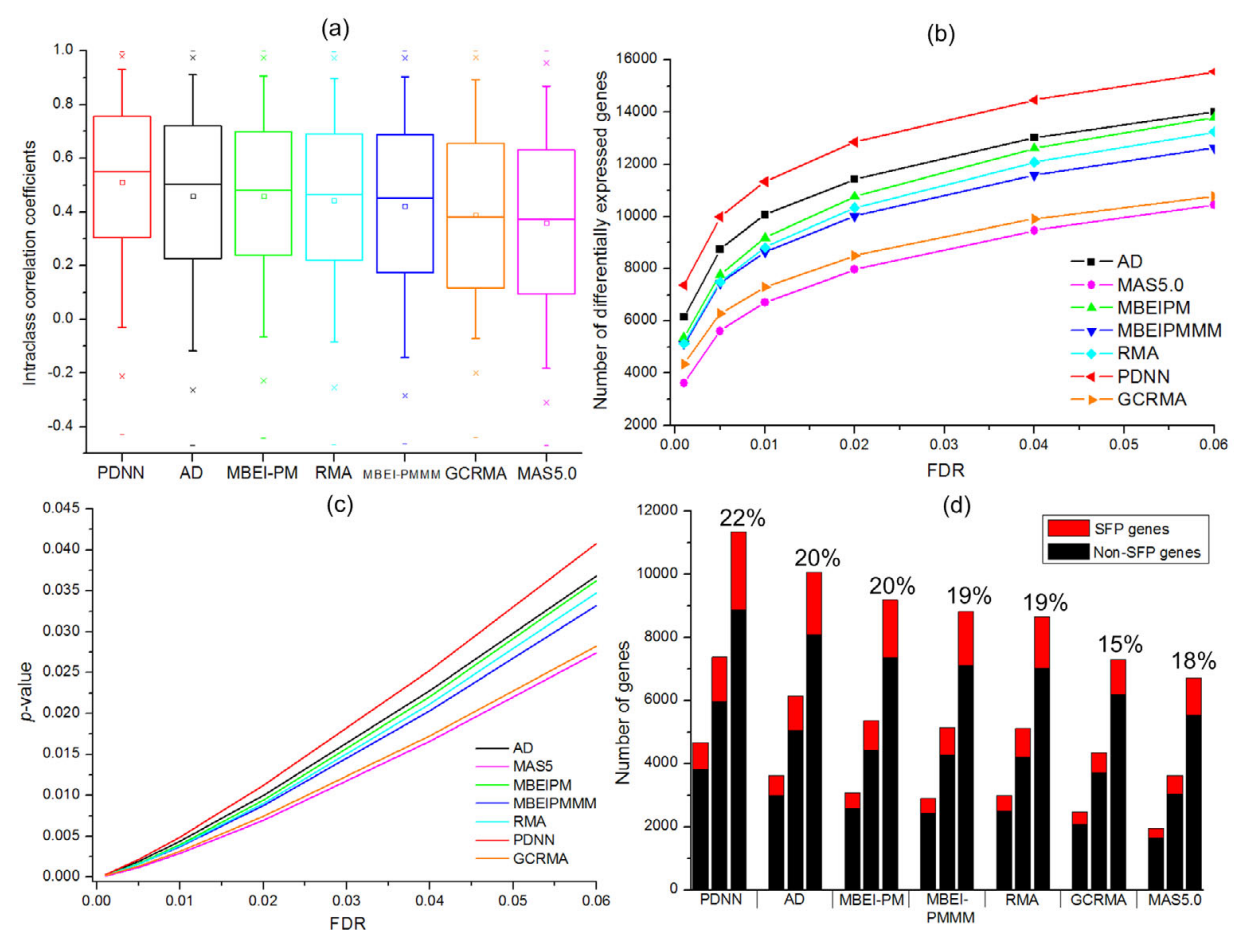
**Statistical properties of estimated barley gene expression indices from seven data extraction methods**. (a) Intraclass correlation coefficients between biological replicates of the estimated expression indices for 22,840 genes; (b) Sensitivity for detecting differentially expressed genes; (c) Calibration *p*-values across FDR levels; and (d) The number of differentially expressed SFP genes (red segment) and non-SFP genes (black segment). For each method the three columns from left to right correspond to FDR levels 0.0001, 0.001 and 0.01. The proportion of genes declared differentially expressed that showed SFP is illustrated for FDR level 0.01.

We compared the ability of each method to calculate consistent gene expression values between biological replicates of a given barley variety using the intra-class correlation coefficients. The box plot in Figure 1a clearly shows the PDNN method gave a superior performance (largest mean and smallest standard deviation) over all of the other methods across all 22,840 genes (*p*-value < 0.0001, Mann-Whitney U-test), while the poorest performers were the GCRMA and MAS5.0 methods (*p*-value < 0.0001, Mann-Whitney U-test). The standard deviation obtained using the PDNN method was significantly lower than all other methods (Levene's test, *p*-value < 0.0001), except MBEI PM for which a similar standard deviation was obtained. In the analysis of the yeast dataset, the PDNN method also gave a superior performance over several of the other methods (Mann Whitney U-test, *p*-value < 0.05, with MBEI methods; *p*-value < 0.0001 with MAS5.0 and GCRMA methods) as shown in Figure 2a [see Additional file 2].

To explain the different performances of the methods illustrated above, we investigated the effect of each step in processing the microarray datasets on estimates of the expression indices in the barley dataset. We tested use of different background correction methods but the same normalization and summarization steps in estimating the genome-wide gene expression indices, and calculated the correlation coefficient for each pair-wise comparison of background correction methods. The correlation coefficients for the MAS5.0 and RMA methods based on different background corrections were greater than 99% and 90%, respectively, which is greater than the correlation between different methods (60%–80%). Therefore the background correction methods did not have a significant effect on the correlation between methods. Similarly, the correlations for the AD and MBEI-PM methods based on different normalization methods were greater than 97% and 99% respectively, showing the normalization methods did not have a detectable effect on the correlation between methods either (Table 5, [see Additional file 3]).

### Efficiency in detecting differential gene expression

To compare the ability to detect differentially expressed genes among the barley varieties for the seven data extraction methods, our primary focus is sensitivity, defined as the total number of genes detected with significant differential expression at a given FDR level. Figures 1b and 2b [see Additional file 2] show the number of genes with significant differential expression called by the seven methods across a range of FDR levels, for the barley and yeast datasets respectively. The numbers of genes declared differentially expressed decreased for each method as the FDR level became more stringent; the best performer at every FDR level was the PDNN method and the worst two performers were GCRMA and MAS5.0. Across all FDR levels, there was marked variation among the seven methods in the number of genes detected as differentially expressed. In particular, PDNN detected 70% more differentially expressed genes than MAS5.0 at FDR 0.01 in both the barley and yeast datasets, and over twice as many genes at even more stringent FDR levels in the barley dataset.

The variation in FDR across the seven methods occurs for two reasons; firstly, variation in the number of genes detected significantly differentially expressed among the varieties and secondly, variation in the expected number of genes with detected significant differential expression when there is no real differential expression. Shedden et al. [16] have shown that different methods differ markedly in their tendency to produce outlier expression values and this is reflected in the thresholds required to achieve a specified proportion of false positive calls. Figures 1c and 2c [see Additional File 1] show how the *p*-value threshold required to achieve a given FDR value differs substantially among the seven methods, for both barley and yeast datasets respectively. Notably, Figures 1b and 1c and also Figures 2b and 2c [see Additional file 2] both illustrate exactly the same order of the seven methods, showing that calibration plays an important role in determining sensitivity in detecting differential gene expression.

### Mutual predictability among the seven methods

An important aspect in comparing the different methods would be to compare their ability to detect the same differentially expressed genes, their mutual predictability. Table 3 shows the pair-wise agreement between the methods for the identity of differentially expressed barley genes at FDR = 0.01. The MAS5.0 method shared the fewest calls with the other methods (for example 61 ± 5%), while PDNN had the strongest agreement with the other methods (93 ± 3%); notably, the order of the seven methods for the pair-wise comparison from the strongest to the weakest was PDNN, AD, MBEI (PM only), RMA, MBEI (PM-MM), GCRMA, MAS5.0, consistent with the order shown in Figure 1a. However, all pair-wise comparisons between methods showed that all methods detected differentially expressed genes not detected by the other methods. This suggests that all methods contribute unique but important information on differential gene expression. Interestingly, methods calling similar genes as differentially expressed did not exhibit greater expression similarity. For example, the gene expression index calculated from the MAS5.0 method is highly correlated with the MBEI PM method (r = 0.958), although the MAS5.0 method only detects 57% of the genes called by MBEI PM at FDR = 0.01. On the other hand, the expression index from MAS5.0 has a much lower correlation (r = 0.581) with that from GCRMA even though the MAS5.0 method calls 75% of the genes called by GCRMA. The results of the yeast data analysis (Table 6, [see Additional file 4]) show exactly the same ordering of the seven methods as that obtained from the barley dataset.

**Table 3:** Mutual predictability of the number of barley genes declared differentially expressed from seven data extraction methods.

Methods	AD	MAS5.0	MBEI^1^	MBEI^2^	RMA	PDNN	GCRMA
AD	10066	5984(89%)	7951(87%)	8030(93%)	7566(86%)	9269(82%)	6610(90%)
MAS5.0	5984(59%)	6716	5257(57%)	5289(61%)	5614(64%)	6243(55%)	5067(69%)
MBEI^1^	7951(79%)	5257(78%)	9185	7674(89%)	6969(79%)	8206(72%)	6048(83%)
MBEI^2^	8030(80%)	5289(79%)	7674(84%)	8650	6744(76%)	7830(69%)	5946(81%)
RMA	7566(75%)	5614(84%)	6969(76%)	6744(78%)	8824	8419(74%)	6736(92%)
PDNN	9269(92%)	6243(93%)	8206(89%)	7830(91%)	8419(91%)	11339	6994(96%)
GCRMA	6610(66%)	5067(75%)	6048(66%)	5946(69%)	6736(76%)	6994(62%)	7310

The diagonal cells show the number of genes declared from each method respectively at FDR = 0.01. The upper and lower triangles show the numbers and percentages (in parentheses) of the genes declared by method *j *(*j *= 1st,...,7th column) and also by method *i *(*i *= 1st,...,7th row, *i*≠*j*). For example, the 5984 genes in common to AD and MAS5.0 represent 89% of those detected by MAS5.0 but only 59% of those detected by AD.

^1 ^MBEI PM only model

^2 ^MBEI PM-MM model

### An empirical Test for efficiency in predicting true differential gene expression

An important objective was to compare the ability of each method to identify genuine differential expression. To this end, we used a recently identified set of over 7000 barley genes containing single feature polymorphisms that largely represent gene expression markers (GEMs) corresponding to a combination of mainly *cis-*acting expression regulators but also *trans*-acting regulators [22]. On this basis, and in the absence of an expected outcome of the differential expression analysis, we propose that differential expression detected for SFP genes is more likely to reflect true differential expression than for genes that do not contain SFP. Using this criterion we compared each of the seven methods for their ability to detect differential gene expression in the SFP genes (Figure 1d) using the proportion of genes declared differentially expressed that showed SFP. The PDNN method outperformed all other methods (chi-square test, *p*-value < 0.0001 at FDR = 0.01), while the worst two methods were MAS5.0 and GCRMA (chi-square test, *p*-value < 0.0001 and *p*-value < 0.05 respectively at FDR = 0.01; moreover, the performance order from best to worst method matched the orders based on sensitivity, calibration and reproducibility (intra-class correlation) analyses. It should be noted that the SFP analysis does not involve any of the methods under investigation here for quantifying gene expression. Thus, the SFP prediction provides an independent source of information for assessing performance of the methods in detecting differentially expressed genes.

## Conclusion

The development of pre-processing methods for Affymetrix oligonucleotide gene expression data has been an area of active research and has led to the availability of a large and growing toolbox of statistical methods for data extraction. This presents a significant challenge for a researcher wanting to identify the most appropriate method to analyze her/his datasets. The present study examined the effect of different data extraction methods on the detection of differentially expressed genes in a barley Affymetrix oligonucleotide microarray dataset. Seven commonly used data extraction methods were used exactly as recommended by their developers, providing a directly relevant comparison of the methods as they will be used in practice by the majority of users of the software, and thus avoiding the well-known over-training problem associated with calibration datasets. The analysis exploits an extensive genome-wide gene expression dataset from eight barley varieties showing extensive variation at phenotypic, transcriptional and genotypic levels. The presence of three replicates for each variety gave a perfectly balanced experimental design and ideal data structure for the main aims of the present research as well as a high power to detect differentially expressed genes by the analysis of variance.

It is clear from the present study that evaluation of the gene expression index is strongly affected by the data extraction method and this in turn has a strong influence on the ability to detect differential gene expression confidently. The seven commonly used methods can be divided into two groups according to the correlation structure in expression indices. Neither the use of different background correction nor normalization procedures could explain the marked variation in expression values estimated from the different methods, as shown previously [15]. Therefore the differences must be caused by the use of different statistical models to estimate the expression values.

Several studies have systematically compared different data extraction methods using tightly controlled calibration datasets, but in doing so, have restricted the comparison to limited amounts of data generated using a limited number of species and platforms [10,12,13]. On the one hand, use of calibration datasets simplifies the data modelling, but on the other hand it avoids the challenges involved in modelling real data involving a larger number of sources of uncontrolled variability. Different studies using Affymetrix spike-in experimental data have tended to produce inconsistent results [9,12,23], possibly due to hidden contaminates. Moreover, the results often conflict with those based on realistic biological datasets. For example, Rajagopalan [11] concluded that it is inadvisable to use the PM only model for microarray data analysis, whereas the current study has shown comparable performance between MBEI PM-MM and MBEI PM only models across all comparisons, and indeed, the PM only model has a superior performance in calculation of consistent gene expression estimates across replicates of a given barley variety (*p*-value < 0.0001, Mann-Whitney U-test).

The major statistical challenge in using real biological experimental datasets arises from the fact that one cannot know *a priori *whether or not a given gene is truly differentially expressed. Therefore in comparing the sensitivity of each of the seven methods to detect differential gene expression, care and attention must be paid to ensure that detected differences in sensitivity among methods are not due to other factors. The Benjamini and Hochberg [24] false discovery rate (FDR) was used here to control the detection of false positives in a way that was not biased in favour of any particular method.

The seven data extraction methods were explored from several angles, including sensitivity, reproducibility and mutual agreement for the identity of differentially expressed genes. Across a range of FDR levels, the PDNN method had the highest sensitivity to detect differentially expressed genes and this was directly related to the less stringent *p*-value threshold required by this method to declare differential expression for a given FDR level. This explains the excellent agreement observed for the differentially expressed genes with all of the other methods. The reproducibility of results from microarray experiments is a critical issue for data analysis methods. The seven data extraction methods showed varying sensitivities to the inherent biological variation expected within the system; the PDNN method produced the most consistent results across biological replicates, whilst MAS5.0 and GCRMA produced the poorest results.

In the absence of an expected outcome, detection of differential expression within those genes with single feature polymorphism was used to further assess the ability of each method to detect genuine differential gene expression. The set of differentially expressed genes identified by the PDNN method was significantly enriched for SFP genes compared to all other methods, reflecting the fact that the method incorporated the sequence information into its calculation of expression indices. The PDNN method may have the highest accuracy in detecting genuine differential gene expression compared to the other six data extraction methods. The GCRMA and MAS5.0 methods called only half the fraction of differentially expressed genes called by PDNN; however, their caution is unlikely to reflect improved prediction of genuine differentially expressed genes.

Taken together, all comparisons suggest that the PDNN method is superior to its rivals for the detection of differentially expressed genes in the current dataset. In contrast, Shedden et al. [16] showed using two datasets of gene expression profiled in human tissue samples that no single method could be identified with consistently superior performance. However, both GCRMA and MAS5.0 methods performed consistently poorly in comparison to rival methods, in agreement with the findings presented here. To assess the performance of the PDNN method in smaller and more statistically challenging biological datasets, we conducted the same analyses using a genome-wide Affymetrix dataset of gene expression profiled on two divergent yeast strains, each with four biological replicates. This analysis provided only a single degree of freedom for detecting differential gene expression between yeast strains, therefore we did not expect it to be as powerful as the barley data analysis. However, the results were remarkably similar to those obtained in the barley data analysis, further supporting the superiority of the PDNN method over its rivals in detecting differentially expressed genes.

We have only used a parametric ANOVA to detect differentially expressed genes. However, variation due to the use of different test statistics is smaller than variation due to different processing methods [16,17] so we expect these differences to be robust to the use of different statistical tests. The PDNN method identifies 70% more differentially expressed genes than MAS5.0, and moreover, gave a superior performance in all the analyses. Nevertheless, each and every method is expected to call one or more differentially expressed genes not called by the other methods. Therefore even the less sensitive methods may contribute to our understanding of which genes are differentially expressed.

The reason for superior performance of the PDNN method based on the present dataset may lie in its use of the free energy statistical model to detect both the specific and non-specific bindings between probes and their corresponding target transcripts, which may accurately model the physical and chemical aspects of probe binding on Affymetrix microarray chips. This may be considered somewhat surprising given findings that positional dependent effects, but not interactions between bases that are physically close, add significant predictive power for specific signal probe effects [25].

The question arising naturally from the present analysis is that of which is the best method for analyzing Affymetrix gene expression data with a view to identifying differentially expressed genes. However, the present study has considered a selection of highly distinguished approaches for data extraction as applied to a barley genome-wide gene expression dataset and recognizes that a greater number of datasets from both controlled experiments and calibration data will be necessary to answer this question. The method chosen will depend on the particular scientific question the study is designed to address and the priorities involved. For example, given the high number of differentially expressed genes detected in a typical microarray experiment, specificity may be a higher priority than sensitivity and influence the method(s) chosen to analyse the results.

## Methods

### Barley RNA microarray data

The microarray data consisted of three biological replicates (*n *= 1,..., 3) from each of eight genetically divergent barley varieties (*m *= 1,...,8) known as Barke, Golden Promise, Haruna Nijo, Morex, Optic, OWB_D, OWB_R and Steptoe (*k *= 24 samples in total). Total mRNA was extracted from the plant leaves, and then hybridized to a Barley 1.0 Affymetrix microarray GeneChip, which consists of 22,840 probe sets (representing 22,840 genes or ORFs), at the Iowa State University transcriptomics facility. A distributed probe set format array was used to prevent potential local image contamination from completely destroying the data of an entire probe set (PM and MM).

The two yeast strains, whose gene expression data was analysed here, were the two haploid parental lines reported in our previous experimental analysis for the genetic dissection of quantitative trait loci affecting ethanol tolerance in budding yeast [26]. The strains were phenotypically divergent for major fermentation traits and cellular morphology characters. The genome-wide gene expression of the strains was profiled at a steady log-growth stage by using Affymetrix yeast 2.0 GeneChips, consisting of 5,814 probe sets. The microarray data consisted of four biological replicates (*n *= 1,...,4) from each of the two yeast strains (*m *= 1,2), a high performance strain designated *PHO*, and a low performance strain designated *PLO *(*k *= 8 samples in total).

### Analysis methods

The raw signal intensities for each probe set (contained in the CEL files) were analysed by the seven most commonly used data extraction methods (AD, MAS5.0, MBEI PM-MM, MBEI PM only, RMA, PDNN and GCRMA) implemented in the R statistical environment. The relevant software was downloaded from the Bioconductor website [27] to produce the genome-wide gene expression indices.

### Comparing the correlation coefficients between methods and between replicates

For the between method comparison *k *correlation coefficients, *r*_*ijk*_, (*k *= 1, 2,..., 24 for the barley data and *k *= 1, 2,...,8 for the yeast data) were calculated between 22,840 (barley) or 5,814 (yeast) corresponding pairs of gene expression indices calculated by methods *i *and *j *from the *k*^th ^microarray sample. The within method correlation was calculated as *r*_*imn*_, corresponding to correlation coefficients calculated from each possible pairing of the *n *= 3 (barley) or *n *= 4 (yeast) replicates for the *m*^th ^barley cultivar (*m *= 1,...,8) or yeast strain (*m *= 1,2) using method *i*. The correlation coefficients (between methods and within method) were compared using the Mann-Whitney U-test.

### Correlation between methods across all samples

Pearson's correlation coefficient was calculated between all pairs of 22,840 (barley) or 5,814 (yeast) gene expression indices calculated from methods *i *and *j *across all *k *= 24 (barley) or *k *= 8 (yeast) samples (*m *cultivars or strains × *n *biological replicates). The genome-wide gene expression indices and the correlation coefficients were then computed under the scenarios of changing a single step in the three-step normalization procedure (background correction or normalization) whilst maintaining the other two steps the same.

### One-way ANOVA to detect differentially expressed genes

Each of 22,840 barley genes (5,814 yeast genes) was tested for differential expression by one-way analysis of variance by partitioning the total variation in gene expression level into variation between groups (8 barley varieties or 2 yeast strains), denoted by $s_{b}^{2}$ and the variation within groups (between replicates), denoted by $s_{w}^{2}$. The F value can then be calculated according to

(1) $$F=\frac{S_{b}^{2}}{S_{w}^{2}}$$

and the associated *p*-value obtained. The false discovery rate (FDR) was controlled according to the standard method of Benjamini and Hochberg [24].

### Intraclass correlation coefficient

For each of the 22,840 barley genes (5,814 yeast genes), we calculated the intraclass correlation coefficient (*r*) according to the standard method of Snedecor and Cochran [28] as

(2) $$r=(s_{b}^{2}-s_{w}^{2})/\{s_{b}^{2}+(n-1)s_{w}^{2}\}$$

for *n *= 3 (barley) or 4 (yeast) replicates.

### Genes recording Single Feature Polymorphisms (SFPs)

We implemented the method proposed in our previous paper [22] to detect single feature polymorphisms (SFPs) that segregate between the 8 barley genotypes. We identified a total of 7340 gene specific SFPs in the barley genome, which are referred to here as the 'SFP genes'.

## Authors' contributions

ZWL conceived of and designed the study. NJ analyzed the data and performed the statistical analyses. LJL contributed to the data analysis and wrote the manuscript. MJK and TJ participated in the design of the study and data analysis. XH provided the yeast dataset. EP, RW and AD were involved in generating the barley microarray data. All authors read and approved of the final manuscript.

## Supplementary Material

Additional file 1 Pearson's Product Moment Correlation Coefficients among yeast gene expression indices calculated from seven different methods. Click here for file

Additional file 2 Statistical properties of estimated yeast gene expression indices from seven data extraction methods. (a) Intraclass correlation coefficients between biological replicates of the estimated expression indices for 5,814 genes; (b) Sensitivity for detecting differentially expressed genes; and (c) Calibration *p*-values across FDR levels. Click here for file

Additional file 3 Pair-wise Pearson correlation coefficients between all pairs of 24 sets (8 barley cultivars × 3 replicates) of 22,840 gene expression indices calculated from the MAS5.0 and RMA methods with different background correction steps and for the AD and MBEI methods with different normalization steps. Click here for file

Additional file 4 Mutual predictability of the number of yeast genes declared differentially expressed from seven data extraction methods. Click here for file
