# Supplementary material for: Methods for evaluating gene expression from Affymetrix microarray datasets
Source: BMC Bioinformatics. 2008 Jun 17;9:284. doi: 10.1186/1471-2105-9-284 (PMC2442103; doi:10.1186/1471-2105-9-284)
Supplement: Additional file 1 — Pearson's Product Moment Correlation Coefficients among yeast gene expression indices calculated from seven different methods. [file 1471-2105-9-284-S1.doc]

Additional Table 4. Pearson’s Product Moment Correlation Coefficients among yeast gene expression indices calculated from seven different methods.

The upper triangle shows the mean and corresponding standard deviation of 8 correlation coefficients, $r_{ijk}$, (*k* = 1, 2, …, 8). $r_{ijk}$ represents the correlation coefficient between 5,814 corresponding pairs of gene expression indices calculated by methods *i* and *j* from the *k*th yeast microarray sample. The diagonal cells show means and standard deviations of 8 correlation coefficients, $r_{imn}$ (*n* = 1,…, 4 and *m* = 1, 2). For *n* = 1,…,4 $r_{imn}$ corresponds to six correlation coefficients calculated from six possible pairs of replicates for the *m*th yeast strain using method *i*. The lower triangle shows the correlation coefficients between all pairs of 5,814 gene expression indices calculated from methods *i* and *j* across all *k* = 8 samples.

| Method | AD | MAS5.0 | MBEI ^1^ | MBEI^2^ | RMA | GCRMA | PDNN |
| --- | --- | --- | --- | --- | --- | --- | --- |
| AD | **0.983±0.009** | 0.992±0.001 | 0.989±0.001 | 0.996±0.001 | 0.746±0.007 | 0.703±0.007 | 0.850±0.006 |
| MAS5.0 | 0.990 | **0.981±0.009** | 0.983±0.002 | 0.990±0.001 | 0.745±0.012 | 0.699±0.012 | 0.843±0.008 |
| MBEI^1^ | 0.989 | 0.982 | **0.984±0.008** | 0.989±0.001 | 0.730±0.005 | 0.685±0.005 | 0.830±0.005 |
| MBEI^2^ | 0.996 | 0.988 | 0.989 | **0.982±0.009** | 0.759±0.007 | 0.715±0.007 | 0.860±0.006 |
| RMA | 0.746 | 0.743 | 0.730 | 0.759 | **0.980±0.013** | 0.985±0.001 | 0.958±0.001 |
| GCRMA | 0.702 | 0.698 | 0.685 | 0.715 | 0.985 | **0.980±0.013** | 0.940±0.002 |
| PDNN | 0.850 | 0.842 | 0.830 | 0.859 | 0.958 | 0.940 | **0.979±0.016** |

^1^ MBEI PM only model

^2^ MBEI PM-MM model
