# Supplementary figures and images for: Methods for evaluating gene expression from Affymetrix microarray datasets
Source: BMC Bioinformatics. 2008 Jun 17;9:284. doi: 10.1186/1471-2105-9-284 (PMC2442103; doi:10.1186/1471-2105-9-284)

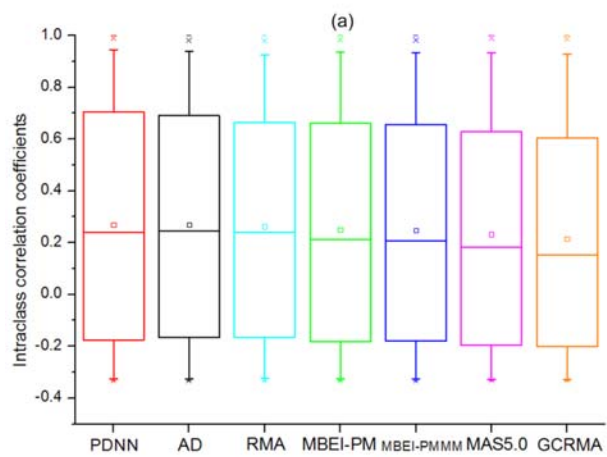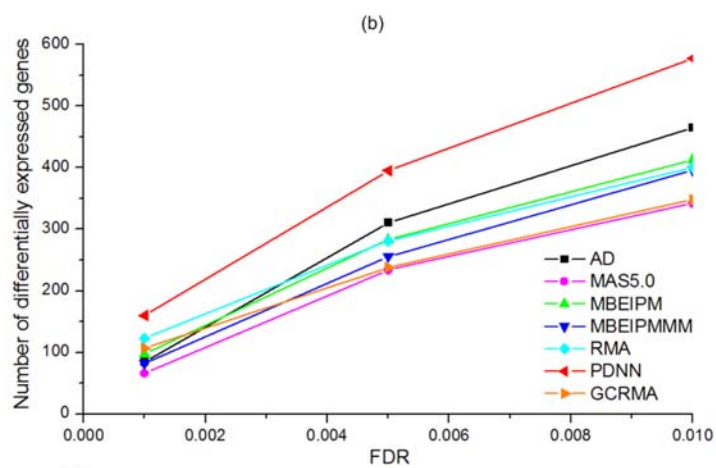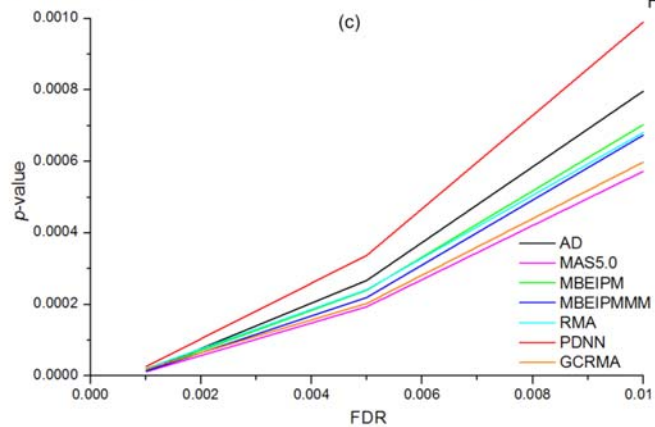

Supplement: Additional file 2 — Statistical properties of estimated yeast gene expression indices from seven data extraction methods. (a) Intraclass correlation coefficients between biological replicates of the estimated expression indices for 5,814 genes; (b) Sensitivity for detecting differentially expressed genes; and (c) Calibration p-values across FDR levels. [file 1471-2105-9-284-S2.pdf]
