# Supplementary material for: Methods for evaluating gene expression from Affymetrix microarray datasets
Source: BMC Bioinformatics. 2008 Jun 17;9:284. doi: 10.1186/1471-2105-9-284 (PMC2442103; doi:10.1186/1471-2105-9-284)
Supplement: Additional file 3 — Pair-wise Pearson correlation coefficients between all pairs of 24 sets (8 barley cultivars × 3 replicates) of 22,840 gene expression indices calculated from the MAS5.0 and RMA methods with different background correction steps and for the AD and MBEI methods with different normalization steps. [file 1471-2105-9-284-S3.doc]

Additional Table 5

Pair-wise Pearson correlation coefficients between all pairs of 24 sets (8 cultivars x 3 replicates) of 22,840 barley gene expression indices calculated from the MAS5.0 (a) and RMA (b) methods with different background correction steps and for the AD method (c) and the MBEI method (d) with different normalization steps. The method used for background correction or normalization accordingly is shown in brackets where this differs from the default method.

(a)

| Method | MAS5.0 | MAS5.0 (None) | MAS5.0 (RMA) |
| --- | --- | --- | --- |
| MAS5.0 | 1 |  |  |
| MAS5.0 (None) | 0.998 | 1 |  |
| MAS5.0 (RMA) | 0.996 | 0.997 | 1 |

(b)

| Method | RMA | RMA  (MAS5.0) | RMA  (None) | RMA  (GCRMA) |
| --- | --- | --- | --- | --- |
| RMA | 1 |  |  |  |
| RMA (MAS5.0) | 0.992 | 1 |  |  |
| RMA (None) | 0.972 | 0.978 | 1 |  |
| RMA (GCRMA) | 0.940 | 0.923 | 0.915 | 1 |

(c)

| Correlation | AD | AD (Quantiles) | AD (Constant) |
| --- | --- | --- | --- |
| AD | 1 |  |  |
| AD (Quantiles) | 0.976 | 1 |  |
| AD (Constant) | 0.972 | 0.996 | 1 |

(d)

| Correlation | MBEI | MBEI (Constant) | MBEI (Quantiles) |
| --- | --- | --- | --- |
| MBEI | 1 |  |  |
| MBEI (Constant) | 0.991 | 1 |  |
| MBEI B (Quantiles) | 0.994 | 0.995 | 1 |
