# Supplementary material for: Methods for evaluating gene expression from Affymetrix microarray datasets
Source: BMC Bioinformatics. 2008 Jun 17;9:284. doi: 10.1186/1471-2105-9-284 (PMC2442103; doi:10.1186/1471-2105-9-284)
Supplement: Additional file 4 — Mutual predictability of the number of yeast genes declared differentially expressed from seven data extraction methods. [file 1471-2105-9-284-S4.doc]

Additional Table 6. Mutual predictability of the number of yeast genes declared differentially expressed from seven data extraction methods.

The diagonal cells show the number of yeast genes declared from each method respectively at FDR = 0.01. The upper and lower triangles show the numbers and percentages (in parentheses) of the genes declared by method *j* (*j*=1st,…,7th column) and also by method *i* (*i*=1st,…,7th row, *i*≠*j*). For example, the 245 genes in common to AD and MAS5.0 represent 72% of those detected by MAS5.0 but only 53% of those detected by AD.

| Methods | AD | MAS5.0 | MBEI^1^ | MBEI^2^ | RMA | PDNN | GCRMA |
| --- | --- | --- | --- | --- | --- | --- | --- |
| AD | 465 | 245(72%) | 352(85%) | 358(90%) | 284(71%) | 376(65%) | 259(74%) |
| MAS5.0 | 245(53%) | 342 | 230(56%) | 225(57%) | 212(53%) | 260(45%) | 197(57%) |
| MBEI^1^ | 352(76%) | 230(67%) | 413 | 351(89%) | 282(71%) | 334(58%) | 255(73%) |
| MBEI^2^ | 358(77%) | 225(66%) | 351(85%) | 396 | 268(67%) | 316(55%) | 246(71%) |
| RMA | 284(61%) | 212(62%) | 282(68%) | 268(68%) | 400 | 361(63%) | 329(95%) |
| PDNN | 376(81%) | 260(76%) | 334(81%) | 316(80%) | 361(90%) | 577 | 322(93%) |
| GCRMA | 259(56%) | 197(58%) | 255(62%) | 246(62%) | 329(82%) | 322(56%) | 348 |

^1^ MBEI PM only model

^2^ MBEI PM-MM model
